# Determination of toxicological relevant arsenic species in urine by hydride generation microwave-induced plasma optical emission spectrometry

**DOI:** 10.1016/j.mex.2024.102893

**Published:** 2024-08-06

**Authors:** Paulina Pizzorno, Lucía Falchi, Nelly Mañay, Mariela Pistón, Valery Bühl

**Affiliations:** a Centro Especializado en Química Toxicológica (CEQUIMTOX), Toxicology Area, DEC, Facultad de Química, Universidad de la República, Gral. Flores 2124, Montevideo, Uruguay; b Grupo de Bioanalítica y Especiación (BIOESP), Analytical Chemistry Area, Facultad de Química, Universidad de la República, Gral. Flores 2124, Montevideo, Uruguay; c Graduate Program in Chemistry, Facultad de Química, Universidad de la República, Gral. Flores 2124, Montevideo, Uruguay; d Grupo de Análisis de Elementos Traza y Desarrollo de Estrategias Simples para Preparación de Muestras (GATPREM), Analytical Chemistry Area, DEC, Facultad de Química, Universidad de La República, Gral. Flores 2124, Montevideo, Uruguay

**Keywords:** Arsenic biomonitoring, Urine, Optic emission spectrometry, Workers' health surveillance, Occupational monitoring, Health risk assessment, HG-MP-AES, Determination of Toxicological relevant species of Arsenic in Urine by Hydride Generation Microwave-Induced Plasma Optical Emission Spectrometry

## Abstract

An analytical method for the determination of toxicological relevant species of arsenic in urine was developed and validated using hydride generation microwave-induced emission spectrometry (HG-MP-AES). This strategy can be used as an alternative to HG-HPLC-ICP-MS considered as a reference technique for arsenic speciation. This procedure is notably less expensive than other techniques and sample preparation and requires only a few steps.

• Hydride generation with MP-AES detection has proven to be an effective technique for measuring arsenic metabolites in urine, which is relevant for occupational monitoring and health risk assessment purposes.
• This method offers simplicity and cost-effectiveness, serving as an alternative to classical analytical procedures typically used for arsenic analysis in urine.
• The methodology has been successfully applied for the purpose of workers' health surveillance.

## Graphical abstract

**Figure:**
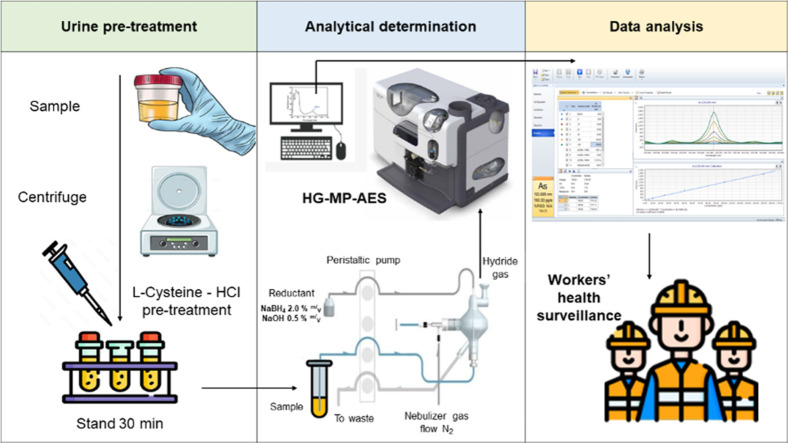

Specifications table

**Table:** 

Subject area:	Chemistry
More specific subject area:	Analytical Toxicology, Monitoring, Health, Environmental
Name of your method:	Determination of Toxicological relevant species of Arsenic in Urine by Hydride Generation Microwave-Induced Plasma Optical Emission Spectrometry
Name and reference of original method:	A simple method for the determination of toxicologically relevant arsenic species in urine by hydride generation microwave-induced plasma optical emission spectrometry for health risk assessment P. Pizzorno, L. Falchi, N. Mañay, M. Pistón, V. Bühl, Spectrochimica Acta Part B: Atomic Spectroscopy 201 (2023) 10,663 https://doi.org/10.1016/j.sab.2023.106630
Resource availability:	Not applicable

## Background

Arsenic, which is highly toxic to humans in its inorganic form, poses a health concern due to occupational exposure to or from natural sources such as contaminated groundwater [1,2]. Therefore, monitoring arsenic exposure from both occupational and natural sources is essential to safeguard public health [3].

Urinary levels of inorganic arsenic and its metabolites, monomethylarsonic acid (MMA) and dimethylarsinic acid (DMA) is widely regarded as the most reliable biomarker of recent exposure, given that the primary route of excretion for inorganic arsenic is through urine [4]. Inorganic arsenic, upon entering the human body through skin contact, inhalation, or ingestion, undergoes methylation reactions that produce MMA and DMA. Subsequently, a mixture containing approximately 10 %–30 % unchanged inorganic arsenic (iAs), 10 %–20 % MMA, and 60 %–70 % DMA is excreted in urine [5,6]. Arsenobetaine, sourced from the diet, particularly from fish and shellfish consumption, has low toxicity and is rapidly absorbed and excreted intact by humans, primarily through urine [5]. However, it is important to note that arsenobetaine does not originate from exposure to inorganic arsenic [4].

Therefore, to assess exposure to iAs, the sum of arsenate, arsenite, DMA, and MMA species in urine is relevant to determine, as recommended, for example, by The American Conference of Governmental Industrial Hygienists (ACGIH) [7]. Arsenobetaine, which originates from the diet, is not quantified because it would result in an overestimation of inorganic arsenic exposure. The sum of arsenate, arsenite, DMA, and MMA species in urine can indeed be referred to as “toxicologically relevant arsenic species”. This term encompasses the primary forms of arsenic that are typically measured in urine to assess exposure and the potential health risks associated with arsenic exposure. By quantifying these specific arsenic species, researchers and health professionals can gain valuable insights into the extent of arsenic exposure and its potential health implications.

Workers in various industries, including agricultural, glass manufacturing, construction, mining, recycling of electronic waste, nonferrous smelting, and woodworkers in countries where CCA (cromated copper arsenate) plants are still active, are at risk of arsenic exposure due to its use in paints, wood preservatives, agricultural chemicals, and glass manufacturing. This underscores the importance of organizations like ACGIH developing Biological Exposure Indices (BEIs®) as guidance values for assessing biological monitoring results. The BEI® generally indicates a concentration below which nearly all workers should not experience adverse health effects. ACGIH has developed a BEI® of 35 µg/L for inorganic arsenic and methylated-arsenic metabolites in urine [7]. Thus, having sensitive, precise, and validated techniques to quantify the abovementioned species of arsenic in urine is critical for monitoring occupational arsenic exposure.

Inorganic arsenic and methylated-arsenic metabolites analysis in urine (As-U) can be performed using a variety of analytical methods [8]. Combining chromatographic separation for each species with spectrometric detection methods is one common strategy. High-performance liquid chromatography (HPLC) separation combined with inductively coupled plasma mass spectrometry (ICP-MS) is the most used technique for speciating arsenic (including arsenobetaine, arsenosugars, among others). This is commonly named as *the gold technique* due to of its excellent separation capabilities, low detection limits, high selectivity, and low matrix interference. Although all these factors make it extremely successful, ICP-MS may not be as widely accessible for regular toxicological investigations in developing countries due to its high costs of operation.

### Method details

We propose a simple alternative method using atomic emission spectroscopy with microwave-induced plasma (MP-AES). This technique allows the coupling of a chamber for hydride generation (HG-MP-AES). This atomic technique uses nitrogen -instead of argon- from a generator powered by compressed air to sustain the plasma. Additionally, and of notable importance, the instrument's investment and maintenance costs are significantly lower than those of any other atomic technique, and it operates without the need for flammable gases in the laboratory [9].

The proposed method for quantifying As-U involves urine sample preparation through a pre reduction step with l-cysteine. This method is based on the reaction of arsenic species with l-cysteine in an acidic medium, producing thio-derivatives that generate arsines at similar rates. This allows the accurate quantification of the total amount of the four toxicologically relevant species (iAs + MMA + DMA) [10,11]. Arsenobetaine, which can lead to an overestimation of iAs exposure as explained before, is not quantified since it does not form hydrides [12,13].

### Chemicals and materials

The glassware was soaked overnight in 10 % (v/v) nitric acid (HNO_3_) (65% w/w, reagent A.C.S., Merck, Darmstadt, Germany) and rinsed with purified water. The purified water was obtained from a Millipore™ Direct Q3 UV water purification system (Millipore, Bedford, MA, USA) (ASTM type I, 18.2 MΩ.cm resistivity). Reagents of analytical grade or higher quality were used. Arsenic stock standard solution (1000 mg *L*^−1^, traceable to SRM from NIST, H_3_AsO_4_ in HNO_3_ 0.5 mol *L*^−1^) was obtained from Sigma Aldrich (Darmstadt, German) and diluted as necessary to obtain calibrators and quality control samples (QC). A 2000 µg *L*^−1^ arsenic intermediate standard was prepared daily by proper dilution with purified water. The stock solutions of As(III) (As(III)-oxide Sigma-Aldrich), MMA (> 97.5 %, Chem Service), and DMA (cacodylic acid >99 %, Sigma-Aldrich) were prepared monthly and kept refrigerated at 4 °C. All arsenic (species) solutions were stable under these conditions when tested after one month. The As(III) stock standard solution 1000 mg *L*^−1^ was prepared by dissolving an appropriate amount of As(III)-oxide in 5 mL of sodium hydroxide 2 M, neutralized with HCl 2 M and diluting with purified water. DMA and MMA stock standard solutions 1000 mg *L*^−1^ were prepared by dissolving an appropriate amount in purified water. An aliquot of those solutions was diluted with water to give the appropriate concentrations of working standard solutions. l-cysteine (>97 %) and sodium tetrahydroborate (NaBH_4_, 99 %) were obtained from Sigma Aldrich (Darmstadt, Germany). Aqueous solutions of l-cysteine 2 % (w/v) in hydrochloric acid solution (HCl) solution 2 % (w/v) were prepared fresh daily. NaBH_4_ 2 % (w/v) in 0.5 % (w/v) sodium hydroxide solution was prepared prior to use. The HCl solution (0.1 mol *L*^−1^) was prepared by diluting appropriate volumes of concentrated HCl (37 % (w/w), reagent A.C.S, J.T. Baker, Mexico City, Mexico) in purified water.

### Instrument

Analytical determinations were performed using an Agilent 4210 microwave-induced plasma atomic emission spectrometer equipped with a standard torch and a Multimode Sample Introduction System (MSIS) for hydride generation (Agilent). Nitrogen was generated with an Agilent 4107 Nitrogen generator (Agilent, Santa Clara, CA, USA), using an air compressor (Dürr Technik, Bietigheim-Bissingen, Germany). The Instrument parameters and operating conditions are shown in Table 1. The operating parameters, such as viewing position and nebulizer flow, were automatically optimized for As with the calibration standard of the maximum concentration by the MP Expert software™ (Agilent) on a daily routine analysis.

**Table 1:** Instrument parameters and operating conditions for arsenic determination using an Agilent 4210 microwave-induced plasma atomic emission spectrometer (MP-AES) system coupled to a Multimode Sample Introduction System (MSIS).

Parameter	Operative condition
As wavelength	188.979 nm
Nitrogen gas source	Agilent 4107 Nitrogen generator
Nitrogen flow rate	0.55 L/min
Microwave frequency	2450 MHz
Applied plasma power	1.0 Kw
Sample introduction system	Agilent MSIS
Sample pump tubing	Black/Black
Reductant tubing	Black/Black
MSIS waste tubing	Black/White
Background correction	Auto
Read Time	10 s
Replicates	3
Pump Speed	30 rpm
Uptake time	0
Stabilization time	10 s
Fast pump during sample uptake	Off
Nebulizer flow	0.95 L/min
Viewing position	30
Calibration fit	Linear

### Sampling and sample preparation

Samples were collected after at least 2 h of retention in urine cups. For workplace monitoring, they were collected with the same retention time at the workplace at the end of a workweek, following the ACGIH guidelines [7].

The specimens were first frozen (<−10 °C) and stored in a portable cooler for delivery to the laboratory. They were analyzed using the proposed procedure by (HG-MP-AES) within 15 days.

### Procedure

#### Pre-reduction step

A mixture of fresh urine (MFU) prepared by combining at least three urine samples obtained from healthy volunteers non-occupationally exposed to As was used to prepare the calibration solutions and the Quality Control (QC) samples. This mixture was stirred for 2 min to homogenize, centrifuged for 15 min at 28,000 *g*, and left to stand at room temperature for 30 min.

The As(V) calibration levels were 0, 10, 25, 50, 75, 100, 150, and 200 µg *L*^−1^. QC samples were prepared in three levels (10, 35, and 100 µg *L*^−1^). Calibrators and QC samples were prepared by transferring 4.00 mL of the MFU into a 10 mL polypropylene tube and adding an appropriate volume of 2000 µg *L*^−1^ As (V) intermediate standard, and then, adding 2 % l-Cysteine solution to a final volume of 8.00 mL. Blanks were prepared by mixing 4.00 mL of the MFU and 4.00 mL of aqueous solutions of l-cysteine. Quantification was performed using matrix-matched calibration, due to the observed significant difference in slope between arsenic (V) standard curves in urine and water [14].

Samples from individuals were processed as follows: Initially, they were stirred for 2 min to homogenize, then centrifuged for 15 min at 28,000 g, and subsequently left at room temperature for 30 to stand at room temperature for 30 min. For further analysis, 4.0 mL of the sample were added to 4.0 mL of l-cysteine solution.

L-Cysteine solution was added simultaneously to calibrators, QC samples and study samples, a critical step of this method. After mixing all tubes by inverting them between 8 and 10 times, they were left to stand at room temperature for 30 min.

The pre-reduction time was determined using the concentrations of reagents previously mentioned by measuring three calibration curves of As(V) prepared in MFU at 0, 50,100,150 µg *L*^−1^, after 15, 30, 60 and 90 min at room temperature. The average slopes of the calibration curves obtained at different times were evaluated. According to Table 2, the slope reached its maximum value at 30 min and remained stable for at least 90 min, indicating that the pre-reduction step requires a minimum resting period of 30 min at room temperature, and the signals are stable for at least 90 min. Subsequently, stability studies were conducted to determine whether this time period could be extended.

**Table 2:** Pre-reduction time and obtained slope values of As(V) calibration curve in MFU with relative standard deviation (RSD).

Pre-reduction time (min)	Slope media (Intensity L / µg) ± RSD% (*n* = 3)
15	11.5 ± 3.1
30	18.1 ± 4.3
60	19.4 ± 3.8
90	18.7 ± 5.0

#### Analytical determination

Hydride generation was carried out with a solution of 2 % NaBH_4_ (w/v) in 0.5 % NaOH (w/v) considering a previous work [15] and the recommendations provided by the MP-AES manufacturer [16].

**Fig. 1:**
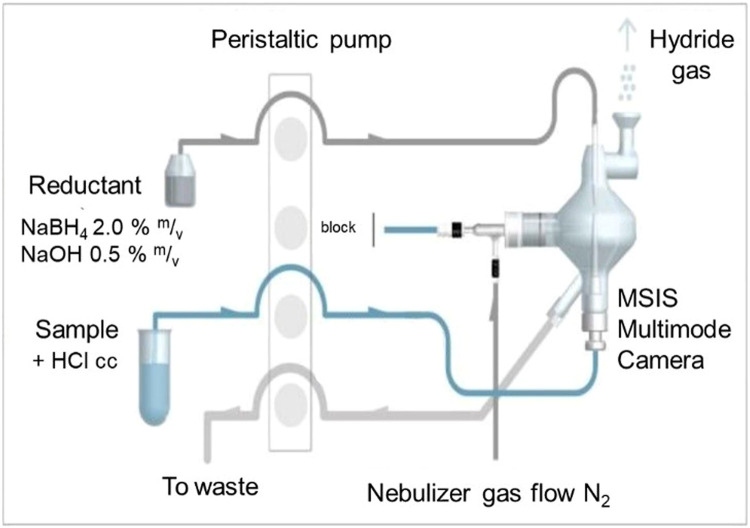
Flow introduction system and MSIS chamber scheme. Adapted from: MSIS – © Agilent Technologies, Inc [16].

Calibrators, QC samples, and samples were introduced one by one via the peristaltic pump into the bottom of the MSIS chamber. Simultaneously, the reductant was introduced through the top of the MSIS. The sample and reductant converged in a thin film that forms at the flow convergence point. The volatile hydride species generated by the reactions were separated from the mixture and carried into the plasma by the nebulizer gas flow (Fig. 1). An aqueous solution of 1 % HCl (v/v) between samples was used to eliminate carryover effect.

### Method validation

Method validation was performed following the recommendations of the Eurachem Guidelines [17]. Some parameters of the Standard Practices for Method Validation in Forensic Toxicology [18], such as stability, were also incorporated since they are useful in laboratory routines, particularly when working with biological samples like urine.

The evaluated figures of merit were linearity, limit of detection (LOD), limit of quantification (LOQ), precision, accuracy, stability, and external proficiency testing control samples.

*Linearity* was calculated with eight calibration levels at concentrations of 0, 10, 25, 50, 75, 100, 150, and 200 µg *L*^−1^. The calibration function was evaluated based on an assay with five replicates of each concentration level. Good linearity was observed, with determination coefficients (R^2^) greater than 0.999 and individual residuals with random distribution were found.

*LOD* and *LOQ* were calculated as 3.3 and 10 times the standard deviation of the measurement blanks (*n* = 15), respectively. The LOD value of 1.8 µg L⁻¹ was comparable to the LOD obtained by our group in previous studies using HG-AAS [15] (1.5 µg L⁻¹) and higher than the 1.0 µg L⁻¹ reported by other groups using HG-AAS [13] and HG-AFS [19]. The lowest LOD reported for arsenic determination in urine samples without coupling with HPLC was obtained by HG-ICP-MS (0.006 µg L⁻¹) [12]. However, the obtained values of *LOD* and *LOQ* were appropriate for use in As biomonitoring applications, excluding the need of expensive equipment.

*Precision and accuracy* were determined using three QC samples at concentrations of 10, 35, and 100 µg *L*^−1^, at low, medium, and high levels, respectively. The QC samples showed acceptable recovery values (Table 3), and the values were within the expected limits (80 %–120 %) [18]. *Intra-assay and inter-assay precision* were evaluated by analyzing the results from five different runs on the same day and were required to be ≤20 % of the coefficient of variation [18]. The *intra-assay* precision obtained for all the studied levels presented a variation of 1.8 %–6.2 %. For the *inter-assay* precision, the mean values ranged from 6.9 % to 15.2 %. These results were within the expected limits.

**Table 3:** Figures of merit for the method validation of the determination of As metabolites in urine by hydride generation microwave-induced plasma optic emission spectrometry (HG-MP-AES).

Linear range (µg *L*^−1^)	LOD (µg *L*^−1^)	LOQ (µg *L*^−1^)	Tested concentrations for QC samples (µg *L*^−1^)	Intra-assay precision (%)	Inter-assay precision (%)	Recovery (%)
5.4 - 200	1.8	5.4	10 (*n* = 15)	6.2	15	95
			35 (*n* = 15)	3.2	6.9	97
			100 (*n* = 15)	1.8	9.0	97

According to the Standard Practices for Method Validation in Forensic Toxicology guidelines, sample *stability* was evaluated under various storage conditions: short-term at room temperature (22 °C); long-term for 15 days at −20 °C; and freeze-thaw stability after undergoing three cycles of freezing and defrosting within 24 h. The results of these evaluations, utilizing three quality control (QC) samples at concentrations of 35 and 100 µg *L*^−1^, shown relative standard deviation (RSD) percentages ranging between 1.9 % and 6.0 %, and accuracy levels within the range of 91 % to 105 %. These findings align with the acceptability criteria outlined in the guidelines [18]. Therefore, these storage conditions are appropriate for sample storage.

Moreover, the stability of the samples after the pre-reduction step was evaluated by measuring medium (35 µg *L*^−1^) and high (100 µg *L*^−1^) quality control (QC) samples at both the initial and 6-hour time points. Achieving a relative standard deviation (RSD%) below 10 % and an accuracy of 103 % for 35 µg *L*^−1^, and 82 % for 100 µg *L*^−1^, reflects favorable outcomes. These findings indicate that samples pre-reduced with l-cysteine can be reliably analyzed within a 6-hour timeframe.

The recovery of As(III), As(V), MMA and DMA was studied to verify that the proposed method is suitable for the quantification of the sum of the four toxicological relevant arsenic species, without overestimating or underestimating any of them. Aliquots of MFU were fortified with each species separately (20 µg *L*^−1^) and with the four species combined (20 µg *L*^−1^ of each species in the same MFU). The recoveries are shown in Table 4, demonstrating good results, ranging between 99 % and 107 % recovery.

**Table 4:** Toxicologically relevant arsenic species recovery.

Species	Spiked Level (µg L⁻¹)	Recovery (%) (*n* = 10)	CV (%) (*n* = 10)
As(III)	20	103	8.4
As(V)	20	107	4.7
MMA	20	107	5.1
DMA	20	111	1.2
As(III)+As(V)+MMA+DMA	20+20+20+20	99	1.8

The analytical method has been evaluated and certified through periodic participation in the German External Quality Assessment Scheme (GEQUAS). The results for the 63–64, 66th and 71st GEQUAS rounds [20] were within the target criteria and acceptable range, and they are shown in Table 5.

**Table 5:** Results for GEQUAS proficiency test (occupational medical field, control urine).

GEQUAS round	Concentration (µg *L*^−1^)	
	Tolerance range *	Result ⁎⁎
63	12.1 – 22.3	12.5
64	21.3 – 37.5	21.4
66	27.7– 40.9	34.4
71	9.8 –18.2	11.9

⁎ Tolerance range provided by GEQUAS-PT.

⁎⁎ Results obtained using the proposed method.

The remaining samples from the GEQUAS proficiency test (PT) were also used as reference materials for internal quality control.

### Determination of as in urine samples

Since 2021, this method has been routinely employed for the assessment of health risks for workers exposed to arsenic. Approximately 100 samples are received annually. In 2023, our laboratory received 85 urine samples from workers at six CCA wood impregnation plants. The urine samples were stored, treated and analyzed using the sample and procedure described above.

The results (Table 6) show that 98 % of the samples had As-U levels below the recommended limit, 58 % of them being below the method quantification limit. Only two of the analyzed samples, showed arsenic levels higher than 35 µg *L*^−1^.

**Table 6:** Results obtained from urine samples of arsenic exposed workers in 2023. The ACGIH limit is 35 µg *L*^−1^.

As-U concentration range (µg *L*^−1^)	Number of samples	Samples%
< 5.0	49	58
≥ 5 – <20	23	27
≥ 20 – <35	11	13
≤ 35	2	2

When a worker's test results indicate elevated values, they must be removed from the workplace for at least 15 days, and a new urine sample must be taken for testing. In this particular case, it was found that upon reanalysis, the values for both workers had returned to safe ranges.

## Conclusion

The proposed analytical method for quantifying the sum of inorganic arsenic and methylated-arsenic metabolites in urine by HG-MP-AES proved to be useful for evaluating occupational health exposure. Additionally, it can also be utilized for evaluating environmental arsenic exposure. Its simplicity is remarkable in providing an alternative to expensive standard analytical techniques for these applications.

## Limitations

Not applicable.

## Supplementary material *and/or* additional information [OPTIONAL]

Data will be available in the PhD thesis of the first author on the public repository of the Universidad de la República: https://www.colibri.udelar.edu.uy/jspui/.

## CRediT authorship contribution statement

**Paulina Pizzorno:** Investigation, Methodology, Writing – original draft. **Lucía Falchi:** Investigation. **Nelly Mañay:** Conceptualization. **Mariela Pistón:** Methodology, Writing – review & editing. **Valery Bühl:** Supervision, Investigation, Methodology, Writing – original draft.

## Declaration of competing interest

The authors declare that they have no known competing financial interests or personal relationships that could have appeared to influence the work reported in this paper.

## Data Availability

Data will be made available on request.
